# The activities of the international precipitation working group

**DOI:** 10.1002/qj.3214

**Published:** 2018-01-15

**Authors:** V. Levizzani, C. Kidd, K. Aonashi, R. Bennartz, R. R. Ferraro, G. J. Huffman, R. Roca, F. J. Turk, N.‐Y. Wang

**Affiliations:** ^1^ CNR‐ISAC Bologna Italy; ^2^ University of Maryland College Park Maryland; ^3^ NASA‐GSFC Greenbelt Maryland; ^4^ Meteorological Research Institute Japan Meteorological Agency Tsukuba Japan; ^5^ Vanderbilt University Nashville Tennessee; ^6^ University of Wisconsin‐Madison Madison Wisconsin; ^7^ NOAA/NESDIS, ESSIC/CICS University of Maryland College Park Maryland; ^8^ OMP/LEGOS Toulouse France; ^9^ Jet Propulsion Laboratory California Institute of Technology Pasadena California

**Keywords:** climate, hydrometeorology, precipitation, rainfall, satellite, snowfall

## Abstract

The International Precipitation Working Group (IPWG) is a permanent International Science Working Group (ISWG) of the Coordination Group for Meteorological Satellites (CGMS), co‐sponsored by CGMS and the World Meteorological Organization (WMO). The IPWG provides a focal point and forum for the international scientific community to address the issues and challenges of satellite‐based quantitative precipitation retrievals, and for the operational agencies to access and make use of precipitation products. Through partnerships and biennial meetings, the group supports the exchange of information on techniques for retrieving and measuring precipitation and for enhancing the impact of space‐borne precipitation retrievals in numerical weather and hydrometeorological prediction and climate studies. The group furthers the refinement of current estimation techniques and the development of new methodologies for improved global precipitation measurements, together with the validation of the derived precipitation products with ground‐based precipitation measurements. The IPWG identifies critical issues, provides recommendations to the CGMS and supports upcoming precipitation‐oriented missions. Training activities on precipitation retrieval from space are also part of the IPWG mandate in cooperation with WMO and other bodies.

## 1 BACKGROUND

Precipitation provides an invaluable freshwater resource for our society and our environment. The availability of freshwater throughout the globe is becoming increasingly important for our agriculture, industry, population and the natural environment, upon which we rely (e.g. UN Environment, 2017; World Water Assessment Programme, 2009). However, the measurement of global precipitation through conventional means is often inadequate due to its coverage being primarily over land, often with poor data latency and poor temporal/spatial scales (Kidd et al., 2017).

Satellite sensors have provided observations for over 40 years, from which precipitation may be derived through exploiting channels in the visible (VIS), infrared (IR) and microwave (MW) spectral bands. The number of available precipitation‐capable sensors has increased over time and currently totals about a dozen, both conical and cross‐track, scanning multichannel passive MW (PMW) instruments, together with geostationary VIS/IR sensors. Despite many of these satellites have exceeded their design lifetime, they still provide useful data thanks to the respective responsible operational agencies. At present, satellite sensors provide global observations at temporal and spatial scales close to many user requirements, complementing surface observations of precipitation that are primarily restricted to populated land regions and (in the case of weather radars) nearby coastal waters. A powerful resource containing the user‐defined requirements for observations and information on all Earth observation satellites and instruments is the Observing Systems Capability Analysis and Review Tool (OSCAR, https://www.wmo‐sat.info/oscar/, last accessed September 21, 2017) of the World Meteorological Organization (WMO).

Commensurate with the availability of satellite observations, a number of techniques have been devised to generate precipitation estimates (e.g. as reviewed in Kidd & Levizzani, 2011; Prigent, 2010). They range from relatively simple schemes, directly relating the satellite observations to surface precipitation, to complex schemes involving radiative transfer modelling to explicitly define the physical processes associated with precipitation. Most schemes utilise multi‐spectral information, while some incorporate multi‐sensor information, both PMW and IR observations, to improve the temporal and spatial resolutions of the final precipitation products. Details on estimating precipitation from space are provided as a community effort in the book edited by Levizzani, Bauer, and Turk (2007).

The first quasi‐global mission dedicated to precipitation and latent‐heat exchanges, the Tropical Rainfall Measuring Mission (TRMM), was launched on November 27, 1997 with a design lifetime of 3 years (Kummerow et al., 2000; Kummerow, Barnes, Kozu, Shiue, & Simpson, 1998; Simpson, Adler, & North, 1988). TRMM operated until 15 April 2015 making it one of the most successful satellites ever launched and provided the first space‐borne Precipitation Radar (PR, Ku‐band). The Global Precipitation Measurement (GPM) core observatory, with its Dual‐frequency Precipitation Radar (DPR, Ka‐ and Ku‐band), was launched on February 27, 2014 and, together with the international GPM constellation, encompasses the current state‐of‐the‐art development in satellite precipitation observations (Hou et al., 2014; Skofronick‐Jackson et al., 2017).

## 2 THE INTERNATIONAL PRECIPITATION WORKING GROUP (IPWG)

### 2.1 Background

The formation of the IPWG was endorsed during the 52nd session of the WMO Executive Council in 2000. The Coordination Group for Meteorological Satellites (CGMS) was encouraged to participate in the formation of the IPWG together with the active participation of the WMO and the Global Precipitation Climatology Project (GPCP: Adler et al., 2003). The International Precipitation Working Group (IPWG) brings together the members of the scientific community to exploit their expertise and expand global precipitation research. The founding meeting of the IPWG was held at Colorado State University in 2001, and was subsequently endorsed by CGMS.

The IPWG is one of five CGMS International Science Working Groups (ISWGs, http://www.cgms‐info.org/index_.php/cgms/page?cat=ABOUT&page=Organisation, last accessed September 21, 2017), the other working groups being the International TIROS Operational Vertical Sounder (TOVS) Working Group (ITWG), the International Radio Occultation Working Group (IROWG), the International Winds Working Group (IWWG), and the International Clouds Working Group (ICWG). Each of these groups provides reports and recommendations to CGMS to further satellite‐based meteorological observations. The main function of the IPWG is to provide a forum for the international scientific community related to operational and research satellite‐based quantitative precipitation estimates. In particular, the IPWG fosters the exchange and promotion of research with the overall aim of improving precipitation products through greater scientific understanding. The group also enables the interests of the precipitation scientific community to be represented at the highest level.

From the onset, the IPWG identified a number of key objectives:

1. Promote standard operational procedures and common software for deriving precipitation retrievals from satellites;
2. Establish standards for validation and independent verification of precipitation estimates;
3. Foster the exchange of data on intercomparisons of operational precipitation retrievals from satellites;
4. Stimulate increased international scientific research and development in this field;
5. Provide recommendations to national and international agencies regarding the utilization of current and future satellite instruments on both polar and geostationary platforms; and
6. Encourage regular education and training activities.

**Figure 1:**
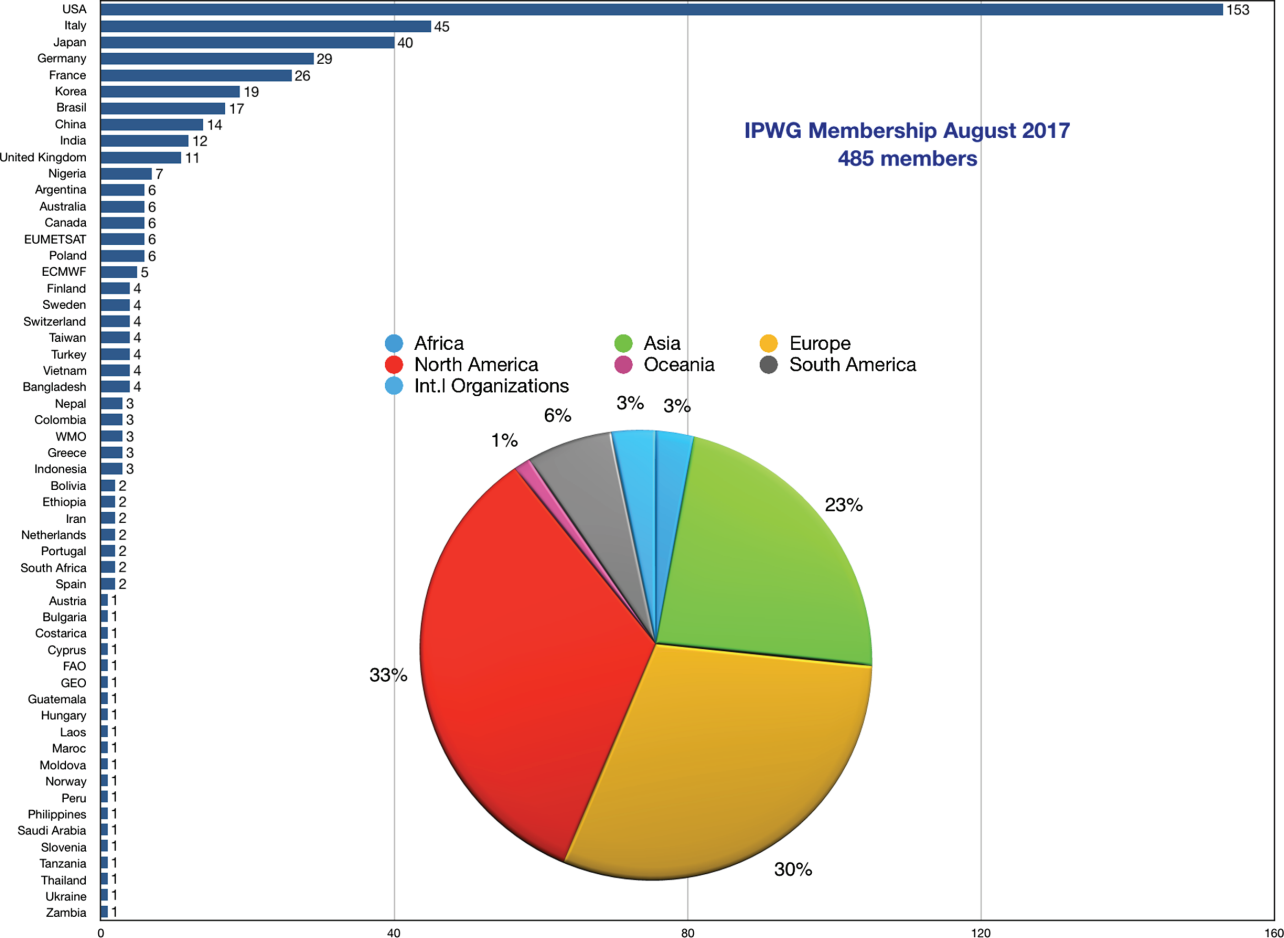
The bar chart represents the number of IPWG members by country/organisation as of July 2017. The pie chart insert shows the distribution of members by region/organisation

Membership is totally free to encourage participation, resulting in a truly worldwide, global group of scientists. Figure 1 shows the membership by country and continent as of July 2017. The IPWG is led by two co‐chairs who oversee the managerial activities of the group, such as the interaction (together with a rapporteur) with the CGMS. The two co‐chairs are selected for a 2‐year term and remain as “outgoing” chairs over the following 2 years to ensure a smooth transition with the new “incoming” co‐chairs.

The IPWG web site (http://ipwg.isac.cnr.it, last accessed September 21, 2017) contains information on the group including datasets, meeting material, reports, access to validation results, and activities of the working groups. Links to relevant IPWG web site pages are listed in Table 1.

**Table 1:** List of relevant pages on the IPWG web site

IPWG home page	http://ipwg.isac.cnr.it/
IPWG datasets	http://ipwg.isac.cnr.it/data.html
IPWG meetings	http://ipwg.isac.cnr.it/meetings.html
IPWG reports	http://ipwg.isac.cnr.it/reports.html
IPWG validation	http://ipwg.isac.cnr.it/calval.html
IPWG working groups	http://ipwg.isac.cnr.it/wg.html

### 2.2 Achievements

Biennial international workshops are organised in order to exchange scientific results and raise relevant issues relating to the observation, retrieval and validation of precipitation. These workshops are hosted at locations around the world to encourage participation from as many nations as possible and have included Madrid (Spain, 2002), Monterey (CA, USA, 2004), Melbourne (Australia, 2006), Beijing (China, 2008), Hamburg (Germany, 2010), São José dos Campos (Brazil, 2012), Tsukuba (Japan, 2014), and Bologna (Italy, 2016). Reports on the activities and outcomes of the workshops have been regularly published in bulletins and journals (e.g. Kidd, Ferraro, & Levizzani, 2010) and are available from the IPWG web site (http://ipwg.isac.cnr.it/meetings.html, last accessed September 21, 2017).

The IPWG has promoted and participated in a number of key meetings, including the World Weather Research Programme (WWRP), World Climate Research Programme (WCRP), GPCP (e.g. 2003, 2005), Global Energy and Water cycle Exchanges (GEWEX), International Geostationary Laboratory (IGeoLab) meetings, National Aeronautics and Space Administration (NASA) GPM mission meetings, Snow Hydrology workshop (e.g. 2008, Colorado), the Program for the Evaluation of High Resolution Precipitation Products (PEHRPP, Geneva, 2007: Turk, Arkin, Ebert, & Sapiano, 2008), the International Workshop on Space‐based Snowfall Measurement (IWSSM; 2005, 2008, 2011, 2013), the Joint Centre for Satellite Data Assimilation (JCSDA) Workshop (Moss Landing, CA, 2016), and the Third Joint JCSDA‐European Centre for Medium‐range Weather Forecasts (ECMWF) Workshop on Assimilating satellite observations of clouds and precipitation from numerical weather prediction (NWP) models (College Park, MD, 2015).

One objective of the IPWG is to provide recommendations to national and international agencies regarding the utilization of current and future satellite instruments. Major contributions have concerned the most prominent precipitation missions of the last two decades, i.e. the US–Japan TRMM (Kummerow et al., 1998, 2000), the French–Indian Megha‐Tropiques (Roca et al., 2015), and the US–Japan GPM (Hou et al., 2014; Skofronick‐Jackson et al., 2017). Moreover, IPWG has provided input to the European Organization for the Exploitation of Meteorological Satellites (EUMETSAT) geostationary and polar programme, in particular the design of the future Meteosat Third Generation (MTG: Stuhlmann et al., 2017; https://www.eumetsat.int/website/home/Satellites/FutureSatellites/MeteosatThirdGeneration/index.html, last accessed September 22, 2017) and the next generation of the EUMETSAT Polar System (Post EPS or EPS Second Generation; https://www.eumetsat.int/website/home/Satellites/FutureSatellites/EUMETSATPolarSystemSecondGeneration/index.html, last accessed September 22, 2017). The same applies to, among others, the Geostationary Operational Environmental Satellite‐R Series (GOES‐R: Kuligowski, Li, Hao, & Zhang, 2016), CloudSat and the A‐Train mission (Stephens et al., 2002, 2017), and the Advanced Microwave Scanning Radiometer for the Earth Observing System (AMSR‐E: Kawanishi et al., 2003) and its follow‐on AMSR‐2 sensor on board the Japanese Global Change Observation Mission‐Water (GCOM‐W: Imaoka, Ito, & Oki, 2010). Crucial interactions have occurred on a regular basis with the agencies that launch and maintain in orbit operational satellites hosting PMW radiometers that are fundamental for precipitation retrieval from space: the US Defense Meteorological Satellite Program (DMSP) Special Sensor Microwave Imager (SSM/I: Hollinger, Peirce, & Poe, 1990) and the Special Sensor Microwave Imager/Sounder (SSMI/S: Kunkee et al., 2008) series extending back to 1987; EUMETSAT's Microwave Humidity Sounder (MHS: Klaes et al., 2007); the Advanced Microwave Sounding Unit‐B (AMSU‐B; Saunders, Hewison, Stringer, & Atkinson, 1995); the Advanced Technology Microwave Sounder (ATMS: Muth, Webb, Atwood, & Lee, 2005) on board Suomi‐NPP (National Polar‐orbiting Operational Environmental Satellite System‐NPOESS Preparatory Project: Lee et al., 2010) and the future US Joint Polar Satellite System (JPSS: Goldberg, Kilcoyne, Cikanek, & Mehta, 2013) series.

The IPWG contributed to two major National Academy of Sciences (NAS) reports (for the TRMM and GPM missions), while an assessment of global precipitation products (Gruber & Levizzani, 2008) was conducted by the IPWG community and the results made available to WCRP. In addition, the group has provided support for the inclusion of the high‐frequency channels (166 and 183 GHz) on the GPM Microwave Imager (GMI), for the implementation of a second Megha‐Tropiques ground station to facilitate better data collection, and for efforts to protect PMW frequency allocations for use in Earth Observation.

### 2.3 IPWG and IWSSM

The most recent (eighth) IPWG meeting was held as a joint meeting with the (fifth) IWSSM meeting in Bologna, Italy. This meeting welcomed 158 participants from 23 countries who contributed 63 oral and 88 poster presentations. The main driving idea on combining the two meetings in one place was to encourage the two communities, which show a substantial degree of overlap, to work together towards a better characterisation of solid precipitation from space.

Indeed, frozen precipitation constitutes a large fraction of all precipitation poleward of about ±60° latitude, yet detection and quantification of snowfall using space‐borne observations pose significant challenges. Levizzani, Laviola, and Cattani (2011) and Bennartz (2014) provide overviews on current issues in space‐based snowfall retrievals. To address this issue, IPWG has supported and endorsed the IWSSM since its inception in 2005 (Bennartz et al., 2011; Bennartz & Ferraro, 2005). IWSSM's main objective is the coordination of international efforts to retrieve falling snow using active and passive microwave instruments. A large component of IWSSM has historically been the evaluation and consolidation of individual ice particle scattering models. At the IWSSM‐5/IPWG‐8 joint meeting in Bologna, Italy, the efforts culminated in the “scattering working group” being established. The main outcomes of the working group were: (a) need for creating a single scattering focus group to discuss formats, share databases and exchange information; (b) single‐scattering properties are just the start of a process towards knowing the bulk scattering and other physical properties of hydrometeors for a better representation of cloud and precipitation physics in models; (c) develop a common scattering database for use in radiative transfer operators; and (d) support full exploitation of precipitation missions through better representation of particle scattering and improved microphysical knowledge. The initiative in Bologna also spawned the 1st International Summer Snowfall Workshop held at the University of Cologne on June 28–30, 2017.

## 3 IPWG WORKING GROUPS AND OUTCOMES

**Figure 2:**
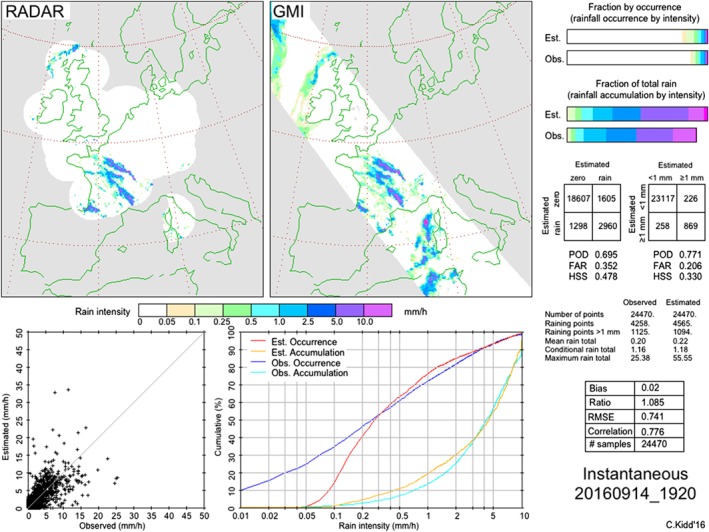
Example of instantaneous intercomparison of precipitation products over western Europe showing scatterplot (lower left), cumulative distribution plot of occurrence and accumulation (lower centre), fraction of coverage and fraction of total rain accumulation (upper right), categorical table of rain/no‐rain (centre right), descriptive statistics and “core” statistics (lower right)

At the biennial workshops, scientists participate in one (or more) of the working groups that have been established by the IPWG to concentrate upon key activities. These working groups currently are:

1. *Validation working group* focuses on the evaluation of a number of research and operational satellite‐based precipitation datasets (further discussed in Section 4). A key activity of this group is sustaining and expanding the intercomparisons of these data with regional verification datasets. While the IPWG has a number of established intercomparison regions (Australia, Europe, Japan, South Africa, South America, USA, see http://ipwg.isac.cnr.it/calval.html, last accessed September 26, 2017) there is a desire to expand these regions to include those with under‐represented meteorological/climatological regimes. The IPWG has recently helped to establish the South African intercomparison region, comparing satellite and surface gauge estimates on a daily basis. Current efforts are helping to establish an intercomparison site over the Indian subcontinent. In addition, intercomparisons of Level2 (swath) GPM precipitation products over western Europe and the USA have been added to assess the performance of techniques at the instantaneous/full resolution scale (Figure 2). As recommended by CGMS at their most recent annual meeting (CGMS‐45, Jeju Island, Korea, June 11–16, 2017, http://www.cgms‐info.org/index_.php/cgms/meeting‐detail/cgms‐45, last accessed September 21, 2017), the IPWG has been tasked to include uncertainty assessments in precipitation retrievals and should be included in future intercomparison work. Furthermore, the validation of snowfall products will be necessary as these become increasingly available. Large‐scale validation should also be encouraged through the use of existing ground validation datasets, such as the Global Precipitation Climatology Centre (GPCC: Becker et al., 2013; Schneider et al., 2016) gauge analysis at daily and monthly scales. The IPWG validation working group reaffirmed the need to maintain *in situ* observations and to develop access to hitherto unavailable surface datasets to ensure the comprehensive validation of satellite precipitation products.
2. *Applications working group* addresses the links between the developers/providers of the precipitation products and the user community. Key issues identified included making links to data and processing/analysis tools available on the IPWG website to better equip users to exploit the satellite‐based precipitation products. In addition, a document/paper providing a review of the suitability and usability of satellite precipitation products was deemed useful to the user community. In connection with the IPWG training efforts an update of the training links on the website was deemed beneficial. The applications group also reaffirmed the need for improved access to ground‐based data in order to improve the satellite‐based precipitation products, and to maintain and coordinate the constellation of precipitation‐capable satellite missions to ensure data availability to the user community. Several applications of satellite‐derived precipitation retrievals have recently emerged in a number of different fields (Kirschbaum et al., 2017; Kucera et al., 2013); IPWG is engaged in expanding them and providing support.
3. *Research working group* focuses upon the development and refinement of satellite‐based techniques, highlighting the need for improved high‐latitude precipitation estimates, identification and retrieval of shallow/orographic precipitation, and better characterization of land surface properties, including surface emissivity and backscattering for improved radiometer and radar precipitation products over land. In addition, the new generation of multi‐spectral VIS/IR sensors on geostationary satellites (e.g. GOES‐16, Himawari‐9, MTG) provide new additional capabilities that should be exploited. This group also identified the need to provide more comprehensive validation of oceanic precipitation to improve retrievals, current validation efforts being essentially (and practically) land‐only. On a programmatic level the group highlighted the need to enhance the spatial and temporal resolution of the observations so that our understanding of microphysical processes may be improved. Multiple and frequent revisit observations, from multi‐ radar or radiometric observations, are encouraged to better understand key cloud–precipitation processes.
4. *Scattering working group* aims to improve the representation of ice‐particle scattering and other physical properties of hydrometeors so that clouds and precipitation can be better represented in radiative transfer models and forward operators. Issues related to the use of scattering properties in precipitation retrieval were initially addressed within the framework of IWSSM. During the 2016 joint IWSSM/IPWG meeting in Bologna, a scattering working group was established as part of IPWG. One outcome of this working group, as well as of earlier IWSSM meetings, was the need for clear definitions of scattering databases or repositories with preferably common data and metadata conventions. Ideally, such scattering databases should be decoupled from radiative‐transfer solvers for flexibility. Further, studies are encouraged to fully describe the microphysics of clouds of different type and geographical areas using existing datasets, including habit descriptions, mixture, mass, size distribution, orientation, phase, degree of riming, fall speeds, as well as these parameters as a function of meteorological parameters. Existing gaps associated with major uncertainties in radiative and microphysical properties can best be addressed with measurement campaigns that offer closure, e.g. airborne radar and passive microwave measurements of radiative properties with co‐located *in situ* measurements (e.g. the GPM Cold Season Precipitation Experiment GCPEX, and the Olympic Mountains Experiment OLYMPEx).
5. *Data assimilation working group* focuses upon the use of the MW observations and precipitation products for NWP and climate models. The group identified the need to hold regular scientific workshops and coordinate research across the CGMS ISWGs for cloudy data assimilation, and in particular to coordinate/develop validation and impact strategies. In particular the group recognised the need to incorporate data assimilation requirements when developing new missions, noting the necessity to sample clouds and precipitation at high temporal and spectral (PMW) resolutions. In addition, an improved latency in the satellite data products is necessary to meet the requirements of the data assimilation community.

The working groups are by no means separate entities. Work on common or overarching issues is encouraged in several ways: (a) the group membership is not fixed and all IPWG members are encouraged to participate in several working groups bringing their own expertise; (b) initiatives stem from the working groups on important issues such as validation exercises and participation in international and/or national activities.

The rapporteur of the IPWG to CGMS works closely with the IPWG co‐chairs and working group chairs to formulate the recommendations from the working group sessions into actionable requests to the CGMS satellite operators. CGMS tracks these items and requests updates from the co‐chairs on an annual basis. Following the IPWG/IWSSM workshop in Bologna a number of recommendations have been made to CGMS centred upon sustaining the precipitation constellation and the need for the formulation of a coordinated plan towards a sustainable PMW‐based constellation (Huffman, Ferraro, Kidd, Levizzani, & Turk, 2016a); accounting for user requirements when specifying instrument temporal/spatial sampling requirements; developing and implementing scattering and emissivity databases; enhancing scientific and operational activities for mixed/solid phase precipitation at mid–high latitudes; and support for training activities (with WMO/CGMS support) on an annual basis.

## 4 IPWG INTERCOMPARISON ACTIVITIES

The IPWG has a number of ongoing activities that provide information and support to the CGMS, such as the generation of white papers on the future of satellite‐based precipitation retrievals (Huffman et al., 2016b). Perhaps the most visible activity is the ongoing intercomparison of satellite‐based precipitation products, which defined a validation “template” that is now used by the international remote‐sensing community for satellite precipitation validation (Ebert, Janowiak, & Kidd, 2007).

A series of intercomparison projects have been carried out in the past to help better understand the quality and utility of satellite precipitation products, ranging from fine‐scale, instantaneous, local comparisons (e.g. AIPs: Ebert et al., 1996), and global, monthly rainfall products (PIP‐1: Barrett et al., 1994; PIP‐2: Smith et al., 1998; PIP‐3: Adler, Kidd, Petty, Morrissey, & Goodman, 2001). These studies targeted specific periods and regions comparing both satellite and model‐based precipitation products against surface reference datasets from both gauges and radars. These studies typically showed a large variation in the performance of the products, although this range was reduced somewhat when more mature products were considered.

The IPWG worldwide validation effort has triggered local studies aimed at characterizing the various precipitation products available at different space and time scales. Kidd et al. (2012) evaluated several high‐resolution precipitation products (HRPP) over northwest Europe and found that satellite products generally exhibit a seasonal cycle in correlation, bias ratio, probability of detection, and false alarm ratio, with poorer statistics during the winter and a general underestimation of precipitation in all seasons. A similar exercise was conducted by Kubota et al. (2009) over Japan showing better performances of the products over ocean, reduced skill over the mountains, and overall poorer results over coastlines and small islands. Turk et al. (2009) and Sohn, Han, and Seo (2010) compared high‐resolution products over the South Korea dense gauge network pointing out the need for accurate PMW measurements as a prerequisite for better estimates by HRPP algorithms. The results of Roca et al. (2010) over West Africa show that the new generation of combined IR–PMW satellite products describes the rain variability similarly to ground measurements while the seasonal variability of the diurnal scale as well as its relative daily importance is only captured by some products. A recent review of HRPP validation was published within the IPWG framework by Maggioni, Meyers, and Robinson (2016).

The GPM Ground Validation (GPM‐GV) programme has organized a series of regionally targeted campaigns aimed at evaluation of GPM precipitation products from precipitation events captured during each specific campaign period. Among them, the Light Precipitation Validation Experiment (LPVEX, Helsinki, Finland, autumn–winter 2010), GCPEX (Ontario, Canada, January–February 2012), the Iowa Flood Season campaign (IFloods, Iowa City, May–June 2013), the Integrated Precipitation and Hydrology Experiment (IPHEx, 2014, focusing on precipitation–hydrological connections), and OLYMPEx (2015, focusing on cold season precipitation and snow from Pacific storm systems). The IPWG intercomparisons complement various other mission‐specific validation efforts by concentrating upon large‐scale validation of the precipitation products through exploiting regional national and international ground‐based observations from gauges or radar. These have been typically performed at 25 km/daily space/time resolutions, although more recently instantaneous 15 km comparisons have started over western Europe. The IPWG regions currently include Australia, Europe, Japan, South Africa, South America, USA. The Megha‐Tropiques Ground Validation (MTGV: Gosset & the French MT Scientific Team and MTGV Team, 2012) programme has demonstrated its validity for a number of applications in satellite rainfall estimation and was proposed as a new IPWG validation site at the 8th IPWG Workshop in Bologna in 2016. Each of the validation sites provides a set of standardized comparisons in near real time (usually <24 hr after production of the products). Figure 2 illustrates a typical output available through the IPWG website: each intercomparison at a minimum contains a scatterplot, categorical table of rain/no‐rain, descriptive statistics and “core” statistics. Additional statistical analysis at the daily or monthly scale is available for some of the regions.

Further intercomparison regions are being encouraged especially in areas where satellite precipitation products are known to have limitations (e.g. orographic, light and solid precipitation).

The IPWG also encourages and supports high‐quality oceanic shipboard precipitation measurements such as those from the Ocean Rainfall And Ice‐phase precipitation measurement Network (OceanRAIN: Klepp, 2015), which are crucial for the improved understanding of hydrological processes as well as for the validation of precipitation over the oceans.

## 5 Outreach

### 5.1 Training

A key activity of IPWG since its inception is the organisation of training sessions on space‐based retrieval of precipitation. Such sessions are regularly organised during the IPWG biennial workshops and represent an integral part of them.

The training component started as a direct contribution to the WMO/CGMS Virtual Laboratory for Education and Training in Satellite Meteorology (VLab, https://www.wmo‐sat.info/vlab/, last accessed September 21, 2017). The first training session was held at the 3rd IPWG workshop in Melbourne (2006) as part of the Asia–Pacific Satellite Applications Training Seminar (APSATS) held in parallel at the Australian Bureau of Meteorology (BoM). Since then, the training sessions have taken place regularly at the workshops in Beijing (2008), Hamburg (2010), São José dos Campos (2012), Tsukuba (2014) and Bologna (2016).

The main aim of the training activities held at the biennial meetings is to involve students and members of operational meteorological centres in the IPWG activities. Scientists and professional teachers from the IPWG community engage in teaching the basic physical and mathematical principles of precipitation retrievals, the structure of the algorithms and the correct way to use them, the use of the available data sets, and the most widespread applications. Attendees vary from PhD students and professional meteorologists, to experts in other disciplines who want to expand their knowledge on precipitation retrieval from satellite observations and how best to utilise them for their own applications.

IPWG has also provided algorithms for use by interested parties. An example is represented by the National Oceanic and Atmospheric Administration (NOAA) SSM/I precipitation algorithm (Ferraro, 1997). The algorithm was made freely available through a simple registration procedure to more than 50 individuals over the past 15 years. IPWG then “mentors” the use of this algorithm through engagement with the requestor to ensure it is being implemented properly.

### 5.2 Support to operational activities

The IPWG started as a CGMS group with operational activities as the fundamental cornerstone. It has since matured/evolved with the division between operations and research transitioned in favour of increasing application areas for research algorithms. The scope of the IPWG has thus broadened to include all of the primary people and affiliated groups in satellite remote sensing of precipitation from space.

However, the operational focus of the group has not disappeared and there are still clear connections with operational institutions and projects around the world. Two examples of these connections are:

- NOAA's Tropical Rainfall Potential (TRaP: Kidder et al., 2005), now Ensemble TRaP (eTRaP: Ebert et al., 2011; http://www.ssd.noaa.gov/PS/TROP/etrap.html, last accessed September 21, 2017), method was presented at the IPWG workshop in Melbourne (2006) and successively was adopted by several agencies in Australia, Korea and Taiwan, who modified it for their own respective operational needs. In fact, the original TRaP was vastly improved to eTRaP as a direct result of engagement between NOAA and BoM at IPWG.
- A strong link exists with the EUMETSAT Satellite Application Facility (SAF) on Support to Operational Hydrology and Water Management (H SAF) for the SAF's precipitation retrieval (Mugnai et al., 2013) and validation activities (Puca et al., 2014). Moreover, H SAF has been a very active member in training activities.

Support to operational activities has now been extended to climate monitoring. The climate services, at least in Europe, have quickly matured in the last few years now reaching an operational phase. These emerging operational activities are supported by a large programmatic framework composed by the European Space Agency (ESA) Climate Change Initiative (CCI: Hollmann et al., 2013), the Copernicus Climate Change Service (C3S, https://climate.copernicus.eu, last accessed September 21, 2017), the EUMETSAT Climate Monitoring SAF (CM SAF: Schulz et al., 2009), the Global Climate Observing System (GCOS: Belward et al., 2016), and by various national programmes. Precipitation is a key parameter in these activities and the IPWG provides the knowledge base on long‐term satellite observations both at the data level as well as at the retrieval level that enables these new services. For instance, the SSM/I Fundamental Climate Data Record (FCDR) efforts (Fennig, Andersson, & Schröder, 2013; Sapiano, Berg, McKague, & Kummerow, 2013) are instrumental in supporting climate monitoring of precipitation and have been discussed in the IPWG community. Similarly, retrievals and merging approaches initiated by the IPWG community are now transferred to operations, e.g. Third Continuous Development and Operations Phase (CDOP‐3) efforts at the CM SAF of EUMETSAT.

New missions dedicated to precipitation are also supported through the IPWG community, in particular, two PMW sensor constellations: the Time Resolved Observations of Precipitation Structure and Storm Intensity with a Constellation of Smallsats (TROPICS: https://tropics.ll.mit.edu/CMS/tropics/Mission‐Overview, last accessed September 21, 2017), and the Temporal Experiment for Storms and Tropical Systems (TEMPEST: Reising et al., 2015) that will benefit from new users thanks to their engagement at the recent IPWG meeting. A further mission, scheduled for launch in March 2018, is the Radar in a CubeSat (RainCube: Haddad, Peral, Tanelli, Sy, & Stephens, 2016; https://www.jpl.nasa.gov/cubesat/missions/raincube.php, last accessed September 21, 2017), a demonstration mission to enable Ka‐band precipitation radar technologies on a low‐cost, quick‐turnaround platform.

## 6 IPWG COORDINATION ACTIVITIES

IPWG is not a governing body and does not have funding authority, being a community working for common goals. However, the group is a formally established CGMS group with specific reporting obligations on its activities. Reporting to CGMS as one of its ISWGs is a guarantee of independence and service to the worldwide community. Through open discussions and meetings, formal documents are produced and formally conveyed to CGMS through the group's Rapporteur. Such documents influence decisions, research and procedures at all levels for ensuring a correct and transparent progress of the discipline, including the technological advances for operational applications worldwide.

IPWG seeks to influence the way precipitation products are used and offers guidance to help informed exploitation of them. One of the most important ways of conveying results and guidance to the users of satellite‐derived precipitation products is the publication of ad hoc studies informing the user on the characteristics of the datasets and reporting the pros and cons together with some guidelines. For example, Kidd, Levizzani, Turk, and Ferraro (2009) addressed the needs of the water management community, while Tapiador et al. (2012) concentrated on global precipitation estimates trying to organize the many links and feedbacks between precipitation measurement, estimation and modelling to identify areas requiring further attention for product development, and to show the limits within which datasets can be successfully used. A similar strategy was recently followed by Tapiador et al. (2017) in indicating paths for the use of global precipitation datasets for climate model validation. Hossain, Biswas, Ashraf, and Bhatti (2017) used the Integrated Multi‐satellitE Retrievals for GPM (IMERG) precipitation data products for flood and irrigation advisories in the Hindu‐Kush region, distributed via mobile phone text messages.

The interaction of IPWG members with other communities has also fostered studies for the development of new multidisciplinary approaches towards the retrieval of precipitation from space. Although the individual efforts are the work of specific groups of people, the IPWG has provided guidance and a testbed to act as a catalyst for such developments, as well as a forum for disseminating datasets and products worldwide. Two examples are represented by the Multi‐Source Weighted‐Ensemble Precipitation (MSWEP) dataset (Beck et al., 2017), which merges gauge, satellite and reanalysis data, and the Soil Moisture to Rain (SM2RAIN) bottom‐up approach (Brocca et al., 2014), which makes use of soil moisture data from satellite to derive precipitation. Both approaches clearly indicate that using data from different platforms is beneficial for better temporal and spatial coverage, while merging data from sources other than satellite can help correct some of the well‐known problems of satellite retrievals. However, care must be taken in the use of, and in the validation of such datasets.

## 7 SUMMARY AND OUTLOOK

The IPWG provides a focus and support for the precipitation science community through a number of activities, including workshops, meetings and education. It encourages the development, exploitation and testing of new techniques and methodologies, together with the intercomparison of precipitation products for operational applications. It also represents the precipitation scientific community, making recommendations to the national and international agencies responsible for overseeing precipitation‐related satellite programmes.

The IPWG has launched a long‐time validation exercise that has encouraged a vast number of individual scientific studies at global and regional levels. The inclusion of new countries and regions will be essential in order to ensure an adequate coverage of precipitation regimes and identify strengths and weaknesses of the retrieval algorithms.

Particular attention is given to supporting upcoming demonstration and constellation programmes for improving both the quality of precipitation estimates and the revisit time for global and regional applications. In this respect, a clear path is necessary to facilitate the improvements of retrieval schemes that need to incorporate new spectral channels and new instruments.

Of particular importance is the outstanding issue of snowfall (e.g. Ebtehaj & Kummerow, 2017; Kneifel, Kulie, & Bennartz, 2011; Kulie et al., 2016; Meng et al., 2017; You, Wang, Ferraro, & Rudlosky, 2017) and hail (e.g. Ferraro, Beauchamp, Cecil, & Heymsfield, 2015; Mroz et al., 2017) estimation as well as the improvement of the retrieval of low‐intensity rainfall (Behrangi, Tian, Lambrigtsen, & Stephens, 2014) and the correction for orographic effects (Shige, Kida, Ashiwake, Kubota, & Aonashi, 2013; Yamamoto & Shige, 2015; Yamamoto, Shige, Yu, & Chen, 2017). In particular, the importance of joint observations of clouds and precipitation has been pointed out by Stephens and Kummerow (2007) to reduce the retrieval errors deriving from the adoption of unrealistic atmospheric models.

The geostationary perspective in rainfall estimation from space will continue to represent an important research and operational topic for the IPWG considering the increasing number of spectral channels of the new sensors launched by several agencies (China Meteorological Administration‐CMA, EUMETSAT, Indian Space Research Organisation‐ISRO, Japan Meteorological Agency‐JMA, Korea Meteorological Administration‐KMA, NOAA, among others) and new instruments that provide potentially relevant ancillary data. An example is given by the new Geostationary Lightning Mapper (GLM: Goodman et al., 2013; http://www.goes‐r.gov/spacesegment/glm.html, last accessed 21 September 2017) on GOES‐R and the upcoming Lightning Imager (LI; https://www.eumetsat.int/website/home/Satellites/FutureSatellites/MeteosatThirdGeneration/MTGDesign/index.html, last accessed September 21, 2017) on MTG; the use of lightning flashes is instrumental in discriminating convective and stratiform precipitation (e.g. Wang, Gopalan, & Albrecht, 2012), improving PMW and IR retrievals (e.g. Grecu, Anagnostou, & Adler, 2000; Xu, Adler, & Wang, 2013), and following the propagation of convective precipitation fields (Dietrich et al., 2011).

These activities will continue to help the progress in the ability to forecast floods and droughts and characterising the Earth water balance (Lettenmaier et al., 2015); for example, accurate precipitation observations from space are a key element of hydrological drought predictions over Africa where *in situ* observations do not have the spatial coverage to estimate the space–time distribution of rainfall (Cattani, Merino, & Levizzani, 2016; Lettenmaier, 2017). At the same time, research is necessary for overcoming the current limitations that still prevent a wider use of satellite precipitation datasets in global and regional hydrology (e.g. McCabe et al., 2017). The IPWG has long since tackled the problem of representativeness and the unconstrained nature of the precipitation retrievals, which suggest a need for combined efforts in remote sensing, modelling and assimilation to improve products (Michaelides et al., 2009).

A careful characterisation of the different satellite‐derived precipitation products is also needed since they have different estimation accuracies depending, among other things, on the physics of the algorithm, the input data, and the differences over sea/land/coast; this is the reason why no unique dataset exists for all applications and why the selection of the most suited precipitation dataset depends on the application itself. For example, hydrological model‐based evaluation strategies are instrumental in identifying the suitability of the various products for hydrology at the catchment level (Behrangi et al., 2011). The increasing number of applications of precipitation products requires that the IPWG supports end users in finding the appropriate datasets and ensuring their correct usage.

## GLOSSARY

**Table:** 

AIP	Algorithm Intercomparison Programme
AMSR‐E	Advanced Microwave Scanning Radiometer for the Earth Observing System
AMSU‐B	Advanced Microwave Sounding Unit‐B
APSATS	Asia–Pacific Satellite Applications Training Seminar
ATMS	Advanced Technology Microwave Sounder
BoM	Bureau of Meteorology (Australia)
CCI	Climate Change Initiative (ESA)
CGMS	Coordination Group for Meteorological Satellites
CMA	China Meteorological Administration
CM	SAF Satellite Application Facility on Climate Monitoring
C3S	Copernicus Climate Change Service
DMSP	Defense Meteorological Satellite Program
ESA	European Space Agency
eTRaP	Ensemble TRaP
EUMETSAT	European Organization for the Exploitation of Meteorological Satellites
FCDR	Fundamental Climate Data Record
GCOM‐W	Global Change Observation Mission‐Water
GCOS	Global Climate Observing System
GCPEX	GPM Cold Season Precipitation Experiment
GEWEX	Global Energy and Water cycle EXchanges
GLM	Geostationary Lightning Mapper
GMI	GPM Microwave Imager
GOES	Geostationary Operational Environmental Satellite
GPCC	Global Precipitation Climatology Centre
GPCP	Global Precipitation Climatology Project
GPM	Global Precipitation Measurement mission
GPM‐GV	GPM‐Ground Validation
HRPP	High‐Resolution Precipitation Product
H SAF	Satellite Application Facility on Support to Operational Hydrology and Water Management
ICWG	International Clouds Working Group
IFloods	Iowa Flood Season campaign
IGeoLab	International Geostationary Laboratory
IMERG	Integrated Multi‐satelliteE Retrievals for GPM
IPHEx	Integrated Precipitation and Hydrology Experiment
IPWG	International Precipitation Working Group
IR	InfraRed
IROWG	International Radio Occultation Working Group
ISRO	Indian Space Research Organisation
ISWGs	International Science Working Groups (CGMS)
ITWG	International TOVS Working Group
IWSSM	International Workshop on Space‐based Snowfall Measurement
IWWG	International Winds Working Group
JCSDA	Joint Centre for Satellite Data Assimilation
JMA	Japan Meteorological Agency
JPSS	Joint Polar Satellite System
KMA	Korea Meteorological Administration
LI	Lightning Imager
LPVEX	Light Precipitation Validation Experiment
MHS	Microwave Humidity Sounder
MSWEP	Multi‐Source Weighted‐Ensemble Precipitation
MTG	Meteosat Third Generation
MTGV	Megha‐Tropiques Ground Validation
MW	MicroWave
NAS	National Academy of Sciences
NASA	National Aeronautics and Space Administration
NPOESS	National Polar‐orbiting Operational Environmental Satellite System
NPP	NPOESS Preparatory Project
OceanRAIN	Ocean Rainfall And Ice‐phase precipitation measurement Network
OLYMPEx	Olympic Mountains Experiment
OSCAR	Observing Systems Capability Analysis and Review Tool
PEHRPP	Program for the Evaluation of High Resolution Precipitation Products
PIP‐x	Precipitation Intercomparison Project‐x
PMW	Passive MicroWave
RainCube	Radar in a CubeSat
SM2RAIN	Soil Moisture to Rain
SSM/I	Special Sensor Microwave/Imager
SSMI/S	Special Sensor Microwave/Imager/Sounder
TEMPEST	Temporal Experiment for Storms and Tropical Systems
TRaP	Tropical Rainfall Potential
TROPICS	Time Resolved Observations of Precipitation Structure and Storm Intensity with a Constellation of Smallsats
TRMM	Tropical Rainfall Measuring Mission
VIS	Visible
VLab	Virtual Laboratory for Education and Training in Satellite Meteorology
WCRP	World Climate Research Programme
WMO	World Meteorological Organization
WWRP	World Weather Research Programme
